# Integrated Transcriptomic and Single-Cell Analyses Identify HILPDA as a Hypoxia-Mediated Regulator of Ferroptotic Signaling in Glioblastoma

**DOI:** 10.3390/ijms27083698

**Published:** 2026-04-21

**Authors:** Nelin Hacioglu

**Affiliations:** Department of Molecular Biology and Genetics, Faculty of Science and Literature, Balikesir University, Çagis Campus, Balikesir 10100, Türkiye; nelinhacioglu@balikesir.edu.tr

**Keywords:** glioblastoma, ferroptosis, hypoxia, HILPDA, tumor microenvironment, iron metabolism, single-cell RNA-seq

## Abstract

Glioblastoma (GBM) is characterized by hypoxia-driven metabolic adaptation and profound therapeutic resistance. Ferroptosis, an iron-dependent lipid peroxidation-related cell death process, has emerged as a potential vulnerability; however, its relationship with hypoxia signaling remains incompletely defined. In this study, we performed integrative transcriptomic and single-cell RNA sequencing analyses to investigate the relationship between hypoxia signaling and ferroptosis-related gene signatures in GBM. Intersection analysis of hypoxia-associated differentially expressed genes and curated ferroptosis-related gene sets identified 29 core candidate genes. FerroScore stratification revealed that tumors with higher ferroptosis-related transcriptional signatures were significantly associated with poor overall survival. Among these genes, HILPDA emerged as a hypoxia-associated gene consistently linked to ferroptosis-related gene expression patterns and immune-related transcriptional programs. HILPDA expression showed significant correlations with iron-ROS axis components, including HMOX1, NOX4, and STEAP3, and was associated with immune microenvironment changes characterized by T cell depletion and inflammatory infiltration. Single-cell RNA-seq analysis further supported the cellular-level association between HILPDA expression and hypoxia-related transcriptional states. Structural equation modeling suggested that the relationship between HILPDA expression and ferroptosis-related gene signatures may be mediated through hypoxia-related pathways. Collectively, these findings indicate a transcriptomic association between hypoxia signaling and ferroptosis-related gene signatures in GBM and identify HILPDA as a candidate gene associated with this axis.

## 1. Introduction

Glioblastoma (GBM) is the most common and aggressive primary malignant brain tumor in adults, accounting for approximately 50–60% of all central nervous system tumors [1]. Classified as stage IV (IDH-wildtype) by the World Health Organization (WHO), this tumor is characterized by its highly infiltrative nature, significant genetic heterogeneity, and treatment resistance [2]. The current standard treatment approach involves maximum surgical resection, followed by radiotherapy and [1,3].

In recent years, ferroptosis has gained significant importance in cancer biology as a form of oxidative cell death characterized by iron dependence and lipid peroxidation [4,5]. Morphologically and biochemically distinct from apoptosis, this process is considered a potential weak point, particularly in treatment-resistant tumor cells [6]. However, the role of ferroptosis in GBM appears to be context-dependent; it interacts with adaptive processes in a hypoxic and metabolically reprogrammed microenvironment [7].

Hypoxia is a key determinant of the aggressive nature of glioblastoma and is commonly observed in the tumor microenvironment [8]. At the heart of this adaptation is hypoxia-inducible factor-1α (HIF-1α), which is stabilized under hypoxic conditions and regulates the transcription of metabolic and antioxidant genes [9]. Specifically, increased SLC7A11 (xCT) expression via the PI3K/AKT/HIF-1α axis can enhance cystine uptake and glutathione (GSH) synthesis, thereby increasing cellular resistance to ferroptosis [10,11]. This mechanism may explain the significant resistance of GBM cells in hypoxic niches to ferroptosis-inducing agents.

The HIF-1 signaling pathway also shapes cellular oxidative stress responses by reorganizing lipid metabolism, iron homeostasis, and reactive oxygen species (ROS) production [12,13]. The expression of iron metabolism-related molecules, such as HMOX1, STEAP3, and ferritin complexes, is directly associated with ferroptotic susceptibility [14]. Furthermore, the hypoxic microenvironment can contribute to immune evasion by suppressing immune cell functions [15]. This suggests a complex cross-talk network among ferroptosis, metabolic reprogramming, and immune suppression.

The literature reports that high expression of ferroptosis regulatory genes, such as SLC7A11, STEAP3, ATF4, and ferritin, is associated with poor prognosis in GBM [16,17]. However, the key transcriptomic nodes that holistically integrate the hypoxia-ferroptosis-immune microenvironment axis are not clear.

In this context, elucidating the integration of hypoxia-sensitive metabolic regulators with ferroptosis-related transcriptional signatures and the immune microenvironment is critical. We aimed to identify candidate genes associated with the hypoxia-ferroptosis axis in glioblastoma by analyzing the intersection of hypoxia and ferroptosis signatures at the transcriptomic level. Based on comprehensive differential expression, enrichment, and correlation analyses, HILPDA, a hypoxia-associated gene, was found to be consistently linked to ferroptosis-related gene expression patterns and immune response programs. Therefore, this study aimed to characterize the transcriptomic relationship between hypoxia signaling and ferroptosis-related gene signatures and to identify candidate genes associated with this axis in glioblastoma.

## 2. Results

To systematically investigate the relationship between hypoxia, ferroptosis, and the immune microenvironment in glioma, we performed a stepwise integrative transcriptomic analysis, progressing from bulk tumor analysis to single-cell resolution.

**Identification of Hypoxia-Associated Ferroptosis Gene Signature in Glioma**

**Figure 1:**
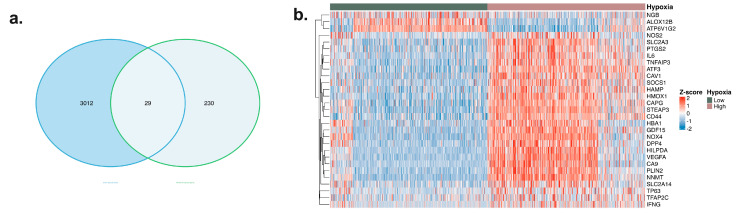

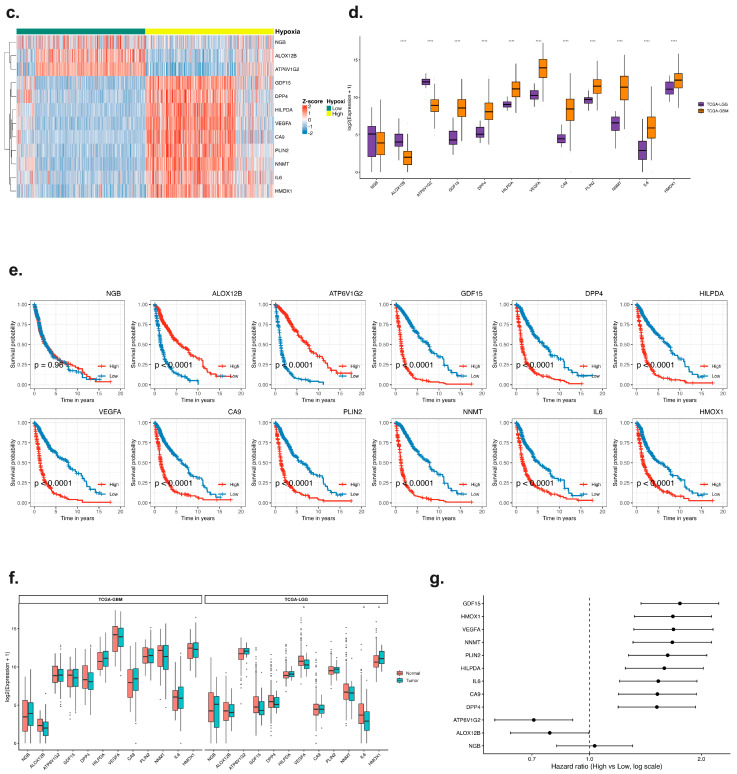
Identification and prognostic evaluation of the hypoxia-associated ferroptosis-related gene signature (TCGA glioma cohorts). (**a**) Overlap between genes differentially expressed under hypoxic conditions and ferroptosis-related genes. Twenty-nine genes located at the intersection of the two gene lists were identified as core candidate genes. (**b**) Expression heatmap of the 29 common genes. Samples were divided into two groups, Low and High, based on the hypoxia score calculated using the ssGSEA method with the HALLMARK_HYPOXIA gene set, with a median cutoff value. (**c**) Expression patterns of prioritized candidate genes from the core signature in the hypoxia-low and hypoxia-high groups. (**d**) Comparison of expression levels of selected candidate genes in the TCGA-GBM and TCGA-LGG cohorts. (**e**) Kaplan–Meier analysis of overall survival according to candidate gene expression levels (high vs. low; median cutoff value). (**f**) Comparison of candidate gene expression levels between tumor and normal tissues in the TCGA cohorts. (**g**) Univariate Cox regression analysis showing the association between candidate gene expression and overall survival; hazard ratio and 95% confidence interval are shown. Statistical significance were indicated as follows: * *p* < 0.05, **** *p* < 0.0001.

To systematically characterize the ferroptosis-related transcriptional changes associated with hypoxia, genes differentially expressed under hypoxic conditions were first compared with the gene set associated with ferroptosis. Twenty-nine common genes located at the intersection of these two lists were identified as core candidate genes representing the transcriptomic overlap between hypoxia and ferroptosis-related gene signatures (Figure 1a). This approach aims to reveal the transcriptomic relationship between the hypoxic microenvironment and ferroptosis-related gene expression programs. The expression profiles of the 29 identified genes were analyzed in samples classified as Low- and High-risk based on the median cutoff value according to the hypoxia score calculated using the ssGSEA method with the HALLMARK_HYPOXIA gene set. Heatmap analysis showed that samples in the hypoxia High-risk group clustered significantly and exhibited a consistent increase in the expression of most of the signature genes (Figure 1b). This suggests that hypoxic signaling is associated with coordinated changes in ferroptosis-related gene expression. When the expression patterns of candidate genes prioritized biologically and prognostically within the core signature were examined, it was observed that the expression levels were more significantly increased in the hypoxia High-risk group (Figure 1c). This finding suggests that hypoxia-related transcriptional programs may be associated with gene expression changes related to lipid peroxidation and iron metabolism. When the distribution of candidate genes among glioma subtypes was evaluated, significant expression differences were found between the TCGA-GBM and TCGA-LGG cohorts (Figure 1d). Specifically, higher expression levels of certain genes were observed in GBM samples associated with a more aggressive clinical course, suggesting that the hypoxia–ferroptosis-related gene signature may be associated with tumor grade. In Kaplan–Meier overall survival analyses based on gene expression levels, high expression of multiple candidate genes was associated with poorer survival (Figure 1e). Conversely, high expression of some genes was associated with better survival, suggesting heterogeneous associations with survival outcomes. No statistically significant survival association was observed for the NGB gene; this indicates that not all hypoxia-related genes are prognostically equivalent. Tumor and normal tissue comparisons revealed that the candidate genes were differentially regulated in tumor tissues (Figure 1f). This result suggests that these genes may be associated with tumor-related transcriptional changes in addition to hypoxia-related responses. Finally, univariate Cox regression analysis quantitatively confirmed the association between candidate gene expression and overall survival at the hazard ratio level (Figure 1g). Genes with HR > 1 showed an increased risk, whereas genes with HR < 1 exhibited a potential protective association. These findings suggest that the hypoxia-associated ferroptosis-related gene signature may be a transcriptomic marker associated with glioma progression.

**Prognostic Significance of Ferroptosis Activity in Glioma**

**Figure 2:**
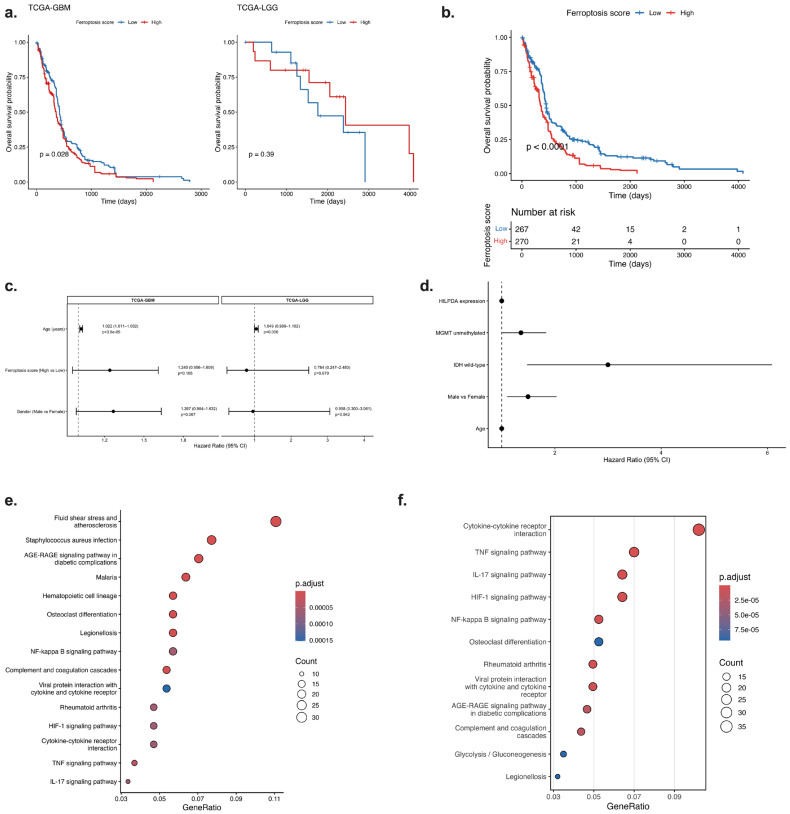
Survival analysis and pathway enrichment analysis of ferroptosis-related transcriptional programs in glioma. (**a**) Kaplan–Meier overall survival analysis based on ferroptosis score in the TCGA-GBM and TCGA-LGG cohorts. High ferroptosis score was associated with significantly worse overall survival in the GBM cohort, whereas no significant difference was observed in the LGG cohort. (**b**) Kaplan–Meier overall survival analysis of all glioma patients stratified by ferroptosis score (high vs. low). Patients with high ferroptosis score showed significantly poorer overall survival. (**c**) Univariate Cox proportional hazards regression analysis showing hazard ratios for gender, age, and ferroptosis score in the GBM and LGG cohorts. (**d**) Multivariate Cox proportional hazards regression analysis in the GBM cohort including HILPDA expression, age, sex, IDH mutation status, and MGMT promoter methylation status. Age, male sex, IDH wild-type status, and unmethylated MGMT promoter were significantly associated with worse overall survival, whereas HILPDA expression was not an independent prognostic factor. (**e**,**f**) KEGG pathway enrichment analysis of ferroptosis-related genes showing significant enrichment in inflammation- and hypoxia-related pathways, including cytokine–cytokine receptor interaction, TNF signaling pathway, IL-17 signaling pathway, HIF-1 signaling pathway, and NF-κB signaling pathway.

To evaluate the relationship between ferroptosis-related transcriptional signatures and glioma prognosis, a FerroScore was calculated for each patient using the GSVA method, and samples were divided into two groups based on the median value: FerroScore-high and FerroScore-low. Kaplan–Meier analyses of the TCGA-GBM and TCGA-LGG cohorts are shown in Figure 2a. In the TCGA-GBM cohort, the FerroScore-high group had significantly shorter overall survival (log-rank *p* = 0.028), whereas in the TCGA-LGG cohort, the survival difference based on the FerroScore was not statistically significant (*p* = 0.39) (Figure 2a). This result suggests that ferroptosis-related gene expression patterns may be associated with prognosis, especially in high-grade gliomas. In a combined glioma analysis created by combining the two cohorts, the FerroScore-high group had significantly worse overall survival (log-rank *p* < 0.0001) (Figure 2b). The risk profile revealed a faster increase in mortality, especially in the early stages, in the high-risk group (Figure 2b). These findings support the possibility that ferroptosis-related transcriptional signatures may be associated with an aggressive disease phenotype.

To evaluate the prognostic value of the FerroScore independent of clinical variables, a univariate Cox regression analysis was performed, and the results are presented in Figure 2c. In the TCGA-GBM cohort, the hazard ratio in the FerroScore-High group was found to be greater than 1 and was associated with a statistically significant increase in risk (Figure 2c). Age emerged as a strong prognostic factor, whereas sex did not show a significant contribution. In the TCGA-LGG cohort, the FerroScore was not found to be an independent risk factor (Figure 2c). These results demonstrate that the ferroptosis-related gene signature is associated with survival outcomes, particularly in the context of glioblastoma. To determine whether HILPDA expression is an independent prognostic factor, multivariate Cox proportional hazards regression analysis was performed in the GBM cohort including age, sex, IDH mutation status, and MGMT promoter methylation status. The multivariate analysis demonstrated that age, male sex, IDH wild-type status, and unmethylated MGMT promoter status were significantly associated with worse overall survival. However, HILPDA expression was not an independent predictor of overall survival in the multivariate model (Figure 2d, Supplementary Table S1).

The genes obtained from the differential gene expression analysis between the FerroScore-high and FerroScore-low groups were subjected to KEGG pathway enrichment analysis, and the results are shown in Figure 2e. Genes associated with the FerroScore-high phenotype were observed to be significantly enriched in inflammatory and stress-response pathways (Figure 2e). Pro-inflammatory pathways, such as the cytokine–cytokine receptor interaction, TNF signaling pathway, IL-17 signaling pathway, and NF-κB signaling pathway, were particularly prominent. Furthermore, the enrichment of the HIF-1 signaling pathway supports the transcriptomic association between FerroScore and hypoxia-related pathways (Figure 2e). The significant involvement of pathways associated with microenvironmental reorganization and chronic inflammation, such as the complement and coagulation cascades and the AGE–RAGE signaling pathway, suggests that FerroScore is associated with inflammatory and hypoxia-related transcriptional programs in glioma.

Overall, when considered together, indicates that ferroptosis-related transcriptional signatures are associated with prognosis, particularly in the glioblastoma subtype, and are transcriptionally associated with inflammatory and hypoxia-related signaling networks. The FerroScore-high phenotype is characterized by increased inflammatory signaling and hypoxia-related transcriptional changes, reflecting more aggressive tumor biology. These findings suggest that the ferroptosis-related gene signature may serve as a transcriptomic biomarker associated with glioma prognosis.

**HILPDA Is Associated with Ferroptosis and Hypoxia Signatures**

**Figure 3:**
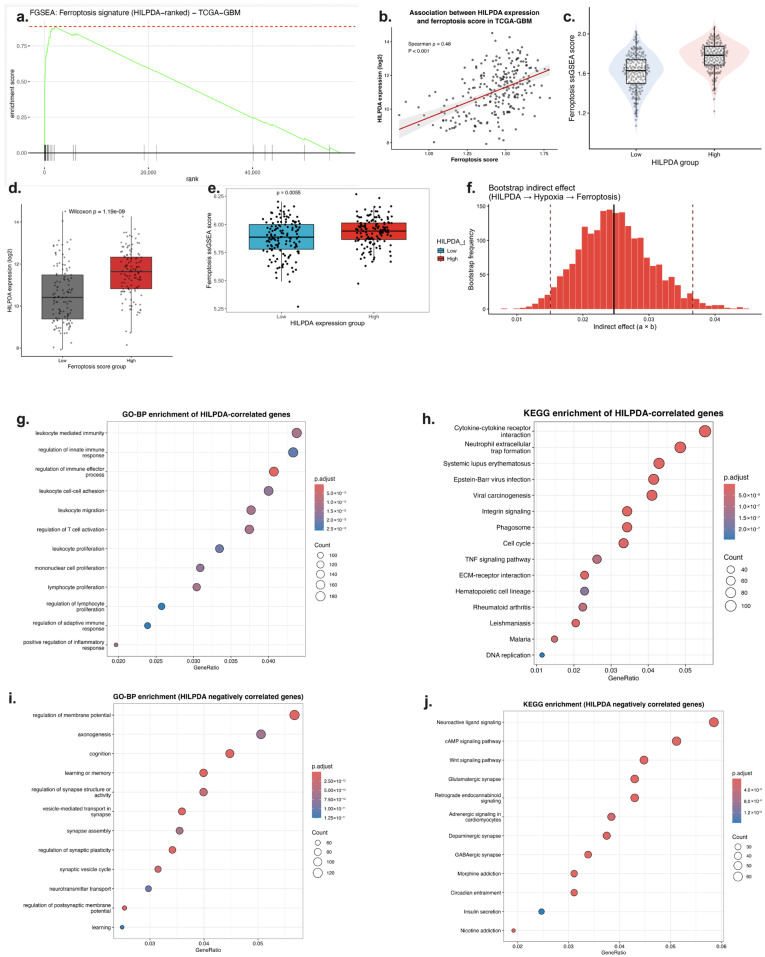
Association of HILPDA expression with ferroptosis-related gene signatures, hypoxia-related transcriptional programs, and associated biological pathways. (**a**) FGSEA analysis of the ferroptosis-related gene signature in the gene list ranked according to HILPDA expression (TCGA-GBM). (**b**) Spearman correlation analysis between HILPDA expression and FerroScore. (**c**) Comparison of HILPDA expression levels in FerroScore-high and FerroScore-low groups. (**d**) Distribution of FerroScore values in HILPDA-High and HILPDA-Low groups. (**e**) Distribution of ferroScore values in HILPDA-high and HILPDA-low groups. (**f**) Bootstrap mediation analysis evaluating whether the association between HILPDA and ferroScore is mediated through hypoxia score. (**g**,**h**) GO-BP and KEGG pathway enrichment analyses of genes positively correlated with HILPDA. (**i**,**j**) GO-BP and KEGG pathway enrichment analyses of genes negatively correlated with HILPDA.

Following the association of ferroptosis-related transcriptional signatures with poor prognosis in glioblastoma, a gene-centric analysis approach was adopted to identify candidate genes associated with this transcriptomic axis. Transcriptomic integration resulted in HILPDA being selected as a prominent candidate gene due to its direct association with hypoxic response and its role in lipid metabolism and stress adaptation processes. FGSEA analysis performed on a gene list ranked according to HILPDA expression revealed significant enrichment of the ferroptosis signature (Figure 3a). This result indicates that elevated HILPDA is associated not only with an increase in individual genes but also with global transcriptional programs related to ferroptosis-associated gene expression.

This observation was confirmed using sample-based analyses. A significant and moderately strong positive correlation was found between HILPDA expression and FerroScore (Spearman’s ρ = 0.48, *p* < 0.001) (Figure 3b). This relationship was also consistent at the categorical level: HILPDA expression was significantly increased in the FerroScore-high group (Figure 3c), and similarly, FerroScore values were significantly higher in the HILPDA-high group (Figure 3d,e). This two-way analysis approach strengthens the statistical consistency of the transcriptomic association between HILPDA expression and ferroptosis-related gene signatures.

Mediation analysis was applied to evaluate whether the association between HILPDA expression and ferroptosis-related gene signatures occurs through hypoxia. The indirect effect calculated using the bootstrap approach in the HILPDA-hypoxia-ferroptosis model was significant (Figure 3g). This finding suggests that the relationship between HILPDA expression and ferroptosis-related gene expression patterns may be mediated through hypoxia-related transcriptional programs. In other words, HILPDA may be associated with hypoxia-related metabolic and transcriptional adaptation rather than directly regulating ferroptosis.

At the functional level, genes positively correlated with HILPDA were found to be significantly enriched in inflammatory and immune response programs (Figure 3h,i). The prominence of pathways, such as cytokine–cytokine receptor interaction, TNF signaling pathway, NF-κB signaling pathway, and HIF-1 signaling pathway, suggests that elevated HILPDA is transcriptionally associated with inflammatory and hypoxia-related pathways. This suggests that ferroptosis-related gene expression changes may be part of broader metabolic and inflammatory transcriptional programs in the tumor microenvironment.

In contrast, genes negatively correlated with HILPDA were enriched in biological processes related to neuronal signaling, synaptic organization, and neurotransmitter activity (Figure 3j,k). This contrasting transcriptional signature suggests that with increased HILPDA, glioma cells may shift towards a less differentiated, more metabolically reprogrammed, and inflammatory phenotype at the transcriptomic level.

Taken together, Figure 3 reveals that HILPDA is a hypoxia-associated gene that is transcriptomically linked to ferroptosis-related and inflammatory gene expression programs. Elevated HILPDA levels are associated with FerroScore and inflammatory signaling pathways via hypoxia-related transcriptional programs. These findings position HILPDA as a candidate biomarker associated with hypoxia–ferroptosis-related transcriptional states in glioblastoma.

**Association of HILPDA with Iron/ROS Pathways and Hypoxia-Related Ferroptosis Signatures**

**Figure 4:**
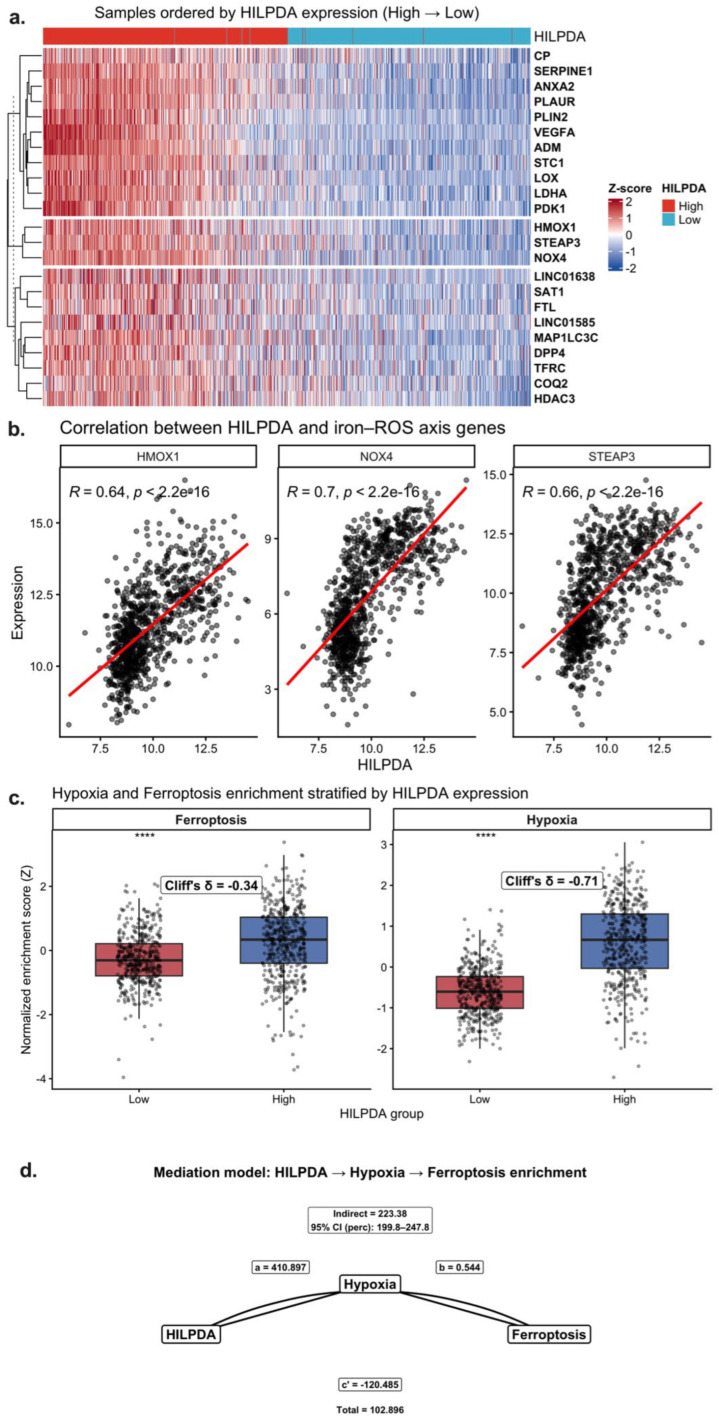
Association of HILPDA expression with iron metabolism, ROS-related pathways, and ferroptosis-related gene signatures via hypoxia-related transcriptional programs (TCGA-GBM). (**a**) Heat map (Z-score) of hypoxia and iron–ROS axis genes in samples ranked according to HILPDA expression. (**b**) Spearman correlation analyses between HILPDA and HMOX1, NOX4, and STEAP3 expression. Red lines indicate linear regression fits. (**c**) Comparison of ferroptosis-related and hypoxia enrichment scores and Cliff’s delta effect size in the HILPDA-High and HILPDA-Low groups. (**d**) Mediation analysis evaluating whether the association between HILPDA and ferroptosis-related gene signatures is mediated through hypoxia score. Statistical significance was indicated as follows: **** *p* < 0.0001.

After demonstrating the correlation between HILPDA and ferroptosis-related gene signatures in previous figures, more targeted analyses were performed to assess whether this relationship occurs via the iron metabolism and oxidative stress axis. Figure 4a presents the expression patterns of genes associated with iron homeostasis, reactive oxygen species (ROS) production, and hypoxic response in samples ranked according to HILPDA expression. Notably, HMOX1, STEAP3, NOX4, and genes associated with lipid metabolism were simultaneously increased in samples with high HILPDA. This gene set is related to iron metabolism and lipid peroxidation pathways, which are biological processes associated with ferroptosis-related gene expression. The heat map shows that high HILPDA is associated with coordinated transcriptional changes along the iron-ROS axis.

Correlation analyses were performed to quantitatively confirm this observation. Figure 4b shows strong and significant positive correlations between HILPDA and HMOX1 (R ≈ 0.64), NOX4 (R ≈ 0.70), and STEAP3 (R ≈ 0.66) (all *p* < 2.2 × 10^−16^). These findings indicate that HILPDA expression is positively associated with genes involved in heme metabolism, iron reduction, and reactive oxygen-related pathways. Therefore, HILPDA appears to be transcriptionally associated with metabolic pathways related to iron metabolism and oxidative stress.

Functional enrichment scores also support this model. Figure 4c shows that both ferroptosis and hypoxia enrichment scores were significantly increased in the HILPDA-High group. According to Cliff’s delta analysis, the effect size for hypoxia was high (δ ≈ −0.71), whereas for ferroptosis it was moderate. This result suggests that increased HILPDA expression is more strongly associated with hypoxia-related transcriptional programs, and that ferroptosis-related gene expression changes may be associated with hypoxia-related transcriptional states.

To further evaluate this relationship, a mediation analysis was performed (Figure 4d). The indirect effect, calculated using the bootstrap approach in the HILPDA → Hypoxia → Ferroptosis model, was found to be significant. The absence of zero in the 95% confidence interval for the indirect effect indicates that hypoxia may mediate the association between HILPDA expression and ferroptosis-related gene signatures. The significant overall effect and prominent contribution of the indirect component support a model in which HILPDA is associated with ferroptosis-related transcriptional changes through hypoxia-related transcriptional programs.

Collectively, Figure 4 reveals that HILPDA is a hypoxia-associated gene that is transcriptionally linked to iron metabolism, oxidative stress pathways, and ferroptosis-related gene expression patterns. Elevated HILPDA levels, together with increased hypoxia-related transcriptional activity, are associated with transcriptional changes in iron metabolism and oxidative stress pathways. This transcriptomic association model suggests that HILPDA may be involved in hypoxia-related metabolic adaptation processes linked to ferroptosis-related gene expression in glioblastoma.

**Association Between HILPDA and the Immune Microenvironment**

**Figure 5:**
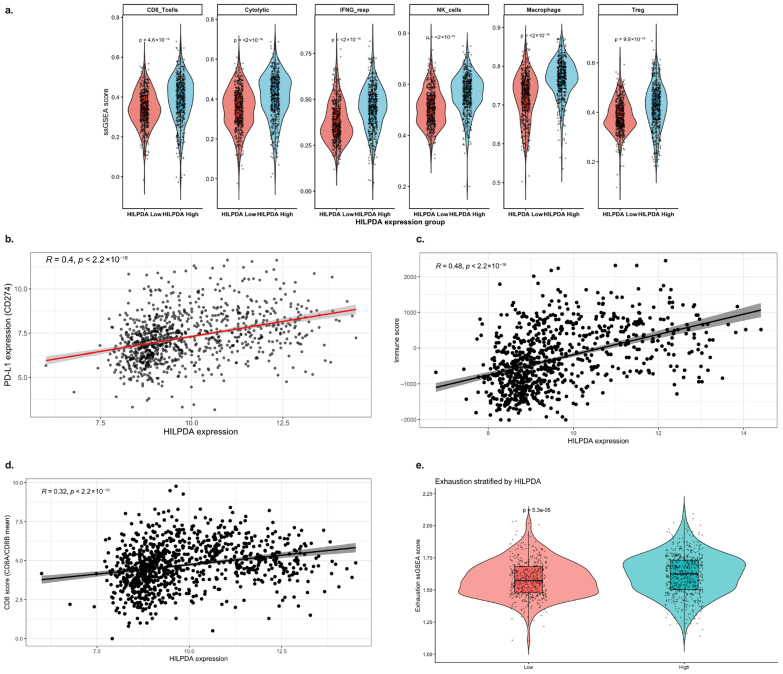
Relationship between HILPDA expression and immune microenvironment signatures. (**a**) Comparison of CD8^+^ T cells, cytolytic activity, IFN-γ response, NK cells, macrophages, and ssGSEA scores in the HILPDA-High and HILPDA-Low groups. (**b**–**d**) Spearman correlation analyses between HILPDA expression and PD-L1 (CD274), IFN-γ response score, and cytolytic activity. Regression lines are shown for visualization purposes; color differences are not indicative of distinct models. (**e**) Comparison of T cell exhaustion scores according to HILPDA expression.

Immune infiltration signature analyses were performed to evaluate the relationship between HILPDA, identified as a hypoxia-associated gene, and the tumor microenvironment. Figure 5a compares immune cell signatures calculated using ssGSEA in the HILPDA-High and HILPDA-Low groups. The CD8^+^ T cell score, cytolytic activity, IFN-γ response, NK cell signature, macrophage infiltration, and Treg score were significantly higher in the HILPDA-High group. This result indicates that higher HILPDA levels are associated with increased immune cell infiltration. However, whether this increase represents a functionally effective antitumor response or a chronic inflammatory microenvironment was evaluated through further analysis. In this context, correlation analyses were performed. Figure 5b shows a significant and positive correlation between HILPDA and PD-L1 (CD274) expression (R ≈ 0.4, *p* < 2.2 × 10^−16^). This finding indicates that elevated HILPDA levels are associated with higher expression of immune checkpoint-related genes.

Figure 5c shows the relationship between HILPDA and IFN-γ response scores. The significant positive correlation revealed that elevated HILPDA was associated with an inflammatory cytokine response. Similarly, Figure 5d shows a positive correlation between cytolytic activity scores and HILPDA. This suggests that HILPDA is associated with an immune-mediated inflamed tumor phenotype. However, increased infiltration alone does not necessarily imply a functional antitumor response. Therefore, the T cell exhaustion signature was evaluated. Figure 5e shows that the exhaustion ssGSEA score was significantly higher in the HILPDA-high group. This finding suggests that elevated HILPDA is concurrent with functional exhaustion and immunosuppression-related transcriptional programs, along with increased immune infiltration.

Collectively, Figure 5 reveals that HILPDA expression is associated with hypoxia-related, ferroptosis-related, and immune-related transcriptional signatures. The HILPDA-High phenotype is characterized by increased IFN-γ response and cytolytic activity, along with concurrent PD-L1 expression and exhaustion programs, which may reflect an “inflamed but dysfunctional” tumor immune microenvironment. These results position HILPDA as a candidate biomarker associated with hypoxia-related and immune-related transcriptional states in glioblastoma.

**Single-Cell Transcriptomic Analysis of the HILPDA–Hypoxia–Ferroptosis Relationship**

**Figure 6:**
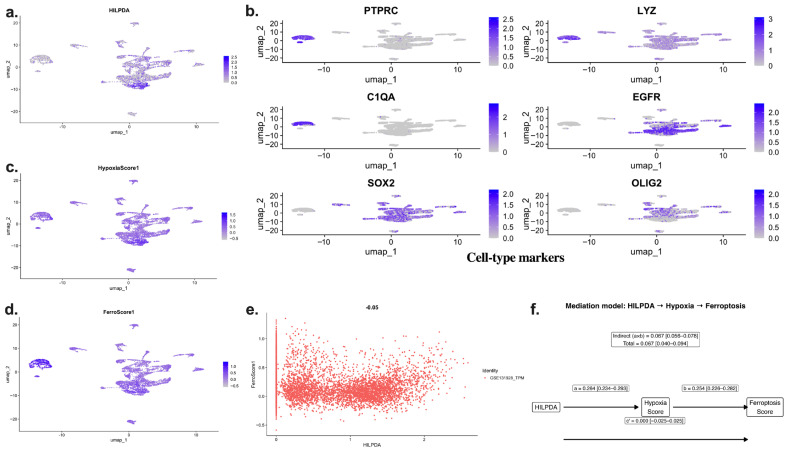
Single-cell transcriptomic characterization of the relationship between HILPDA expression, hypoxia score, and ferroptosis-related gene signatures. (**a**) Distribution of HILPDA gene expression on a UMAP projection of the GSE131928 single-cell glioma dataset. (**b**) UMAP expression maps for immune (PTPRC, LYZ, C1QA) and tumor/stem (EGFR, SOX2, OLIG2) cell markers. (**c**) Distribution of HypoxiaScore1 on a UMAP projection. (**d**) Distribution of FerroScore1 on a UMAP projection. (**e**) Distribution plot showing the relationship between cell-based HILPDA expression and FerroScore1. (**f**) Structural equation modeling (SEM) analysis evaluating whether the association between HILPDA expression and ferroptosis-related gene signatures is mediated through hypoxia score.

Single-cell RNA-seq analysis (GSE131928) revealed that HILPDA expression was concentrated in specific tumor cell subpopulations along the UMAP projection (Figure 6a). Marker genes used for cellular annotation (PTPRC, LYZ, C1QA, EGFR, SOX2, and OLIG2) showed that HILPDA expression was particularly enriched in tumor/stem-like cell clusters (Figure 6b). HypoxiaScore1, calculated in the same cells, showed significant overlap with HILPDA-high cellular niches (Figure 6c), whereas FerroScore1 showed an increased trend in hypoxic cellular subpopulations (Figure 6d).

Cellular-level correlation analysis revealed a positive and significant association between HILPDA and HypoxiaScore1 (Spearman ρ ≈ 0.19, *p* < 2.2 × 10^−16^). However, the direct correlation between HILPDA and FerroScore1 was weak and borderline (ρ ≈ 0.02, *p* = 0.059), suggesting that the relationship may be indirect and potentially linked to hypoxia-related transcriptional programs.

To further evaluate this relationship, a structural equation modeling (SEM) mediation analysis was performed. The effect of HILPDA on HypoxiaScore1 was significant (β = 0.264, 95% CI: 0.234–0.293, *p* < 0.001), and the effect of HypoxiaScore1 on FerroScore1 was also significant (β = 0.254, 95% CI: 0.226–0.282, *p* < 0.001). The direct effect of HILPDA on FerroScore1 was not significant (c′ = 0.000, *p* = 0.987). In contrast, the indirect effect (a × b) was significant (β = 0.067, 95% CI: 0.056–0.078, *p* < 0.001), and the total effect was largely explained by this indirect component (Figure 6f).

These findings suggest that the association between HILPDA expression and ferroptosis-related gene signatures at the single-cell level may be linked to hypoxia-related transcriptional activity rather than a direct association. Overall, the single-cell analysis supports the transcriptomic association model observed in bulk RNA-seq analyses and indicates that HILPDA-high cells are located in hypoxia-associated cellular niches that also exhibit ferroptosis-related gene expression patterns.

## 3. Discussion

This study systematically analyzed the intersection of hypoxia-dependent transcriptional programs and ferroptosis-related gene networks in gliomas. The integration of bulk RNA-seq data, pathway enrichment analyses, immune microenvironment assessment, and single-cell RNA-seq modeling revealed that HILPDA may be a candidate gene associated with hypoxia-related and ferroptosis-related transcriptional signatures. Our findings suggest that ferroptosis-related transcriptional signatures, particularly in the glioblastoma (GBM) subtype, may be associated with hypoxia-dependent metabolic and inflammatory reprogramming.

Ferroptosis is a regulated form of cell death based on iron-dependent lipid peroxidation [4]. In recent years, the role of ferroptosis in cancer biology has been extensively investigated and considered a potential therapeutic weakness, particularly in treatment-resistant tumor cells [6]. However, the effect of ferroptosis in the tumor context is not one-sided. Chronic metabolic stress and hypoxic adaptation have been reported to reprogram the ferroptotic process and may be associated with tumor progression, depending on the context [16]. The finding in this study that the FerroScore-High group was associated with significantly poorer survival in the GBM cohort suggests that ferroptosis-related transcriptional signatures may be associated with an aggressive tumor phenotype. This finding suggests that ferroptosis-related transcriptional programs may be associated with tumor adaptation processes rather than directly indicating ferroptotic cell death. Therefore, the FerroScore used in this study should be interpreted as a transcriptional ferroptosis-related signature rather than a direct measurement of ferroptotic cell death. Importantly, the ferroptosis score used in this study represents a ferroptosis-related transcriptional signature rather than a direct measurement of ferroptotic cell death. Therefore, the results should be interpreted at the transcriptomic level rather than as a direct indicator of ferroptotic activity.

Hypoxia is one of the most prominent microenvironmental features of glioblastoma and is directly related to tumor aggressiveness [8]. HIF-1α, the main regulator of hypoxic adaptation, activates metabolic reprogramming, iron metabolism, and antioxidant defense systems [13]. The literature has shown that SLC7A11 expression is increased via the PI3K/AKT/HIF-1α axis, and this enhances ferroptosis resistance [11]. In our study, the enrichment of HIF-1 signaling pathways and inflammatory pathways in the FerroScore-High phenotype supports the idea that ferroptosis-related transcriptional programs are associated with the hypoxic and inflammatory microenvironment. This suggests that ferroptosis-related gene expression changes may be part of hypoxia-associated metabolic adaptation in GBM.

Within this regulatory axis, HILPDA is a remarkable molecule. HILPDA (hypoxia-inducible lipid droplet-associated protein) is a hypoxia-sensitive protein regulated by HIF-1α and plays a role in lipid droplet dynamics and energy homeostasis. Because lipid droplets and polyunsaturated fatty acids constitute the main substrates of ferroptosis, the role of HILPDA in lipid metabolism suggests a potential link with ferroptosis-related metabolic pathways. Our data revealed that HILPDA expression was significantly and positively correlated with the ferroptosis score (ρ ≈ 0.48) and was highly parallel to genes, such as HMOX1, NOX4, and STEAP3, located on the iron-ROS axis. In glioma, patient survival is strongly influenced by established molecular markers such as IDH mutation and MGMT promoter methylation status. Therefore, we performed multivariate Cox regression analysis including these major prognostic variables to control for clinical and molecular confounding. The multivariate analysis demonstrated that IDH status, MGMT promoter methylation, age, and sex remained independent predictors of survival, whereas HILPDA expression was not significant in the multivariate model. This finding indicates that HILPDA is not an independent prognostic factor but rather reflects hypoxia-associated tumor biology. Thus, the prognostic association observed in univariate analyses is likely mediated by underlying hypoxia-driven transcriptional programs and tumor molecular subtype. Therefore, HILPDA should be interpreted as a hypoxia-associated biomarker reflecting tumor microenvironmental stress rather than a standalone clinical prognostic marker.

Single-cell RNA-seq analysis confirmed this axis at the cellular level. HILPDA expression was shown to be concentrated in specific tumor cell subpopulations and to significantly overlap with the hypoxia score. Although a positive and significant relationship was found between HILPDA and the hypoxia score at the cellular level, the direct relationship between HILPDA and the ferroptosis score remained weak. Mediation analysis performed using structural equation modeling showed that the relationship between HILPDA expression and ferroptosis-related gene signatures may be mediated through hypoxia-related transcriptional programs. The HILPDA-Hypoxia and Hypoxia-Ferroptosis pathways were found to be significant; however, the direct effect of HILPDA on ferroptosis was insignificant. In contrast, the indirect effect (a × b) was statistically strong, and almost all of the total effect was explained through this mediating pathway. This complete mediation pattern suggests that HILPDA may be associated with hypoxia-related metabolic reprogramming rather than directly regulating ferroptosis. These results support a transcriptional association model rather than a direct regulatory mechanism.

The relationship between HILPDA and the immune microenvironment is noteworthy. Increased CD8^+^ T cell infiltration, cytolytic activity, and IFN-γ response in the HILPDA-High phenotype indicate an inflammatory tumor phenotype. However, increased PD-L1 expression and T cell exhaustion scores suggest that this infiltration may be functionally suppressed. This inflamed but dysfunctional microenvironment model suggests that the hypoxia-ferroptosis axis suggests the presence of a metabolic and immunological transcriptional association pattern. Hypoxia has been shown to contribute to immunosuppression by increasing PD-L1 expression [15], and our findings are consistent with this transcriptomic association. Importantly, these findings reflect transcriptomic associations and do not imply a direct regulatory role of HILPDA in immune modulation, but rather suggest that HILPDA expression is associated with hypoxia-related immunological transcriptional programs.

This study has several limitations. The analyses are primarily based on transcriptomic data and statistical modeling; therefore, the findings should be interpreted as transcriptomic associations rather than mechanistic evidence. The ferroptosis score used in this study represents a ferroptosis-related transcriptional signature rather than a direct measurement of ferroptotic cell death. Although single-cell RNA-seq analysis increased cellular resolution, functional validation experiments, such as HILPDA knockdown/overexpression and ferroptosis sensitivity assays under hypoxic conditions, are required to establish causality. Therefore, this study should be considered hypothesis-generating and provides a framework for future functional studies investigating the hypoxia–ferroptosis relationship in glioblastoma. In addition, although survival analyses suggest a potential prognostic association, glioma prognosis is influenced by multiple molecular and clinical factors, and therefore the prognostic value of HILPDA should be interpreted with caution. It is important to note that GSVA-derived ferroptosis scores do not directly measure ferroptotic activity, which is a dynamic and biochemical cell death process, but rather reflect ferroptosis-related transcriptional signatures. Therefore, our results should be interpreted as transcriptomic associations rather than direct evidence of ferroptosis activation.

In summary, this study provides an integrative transcriptomic framework linking hypoxia signaling, ferroptosis-related gene expression signatures, and immune microenvironment remodeling in glioblastoma. Rather than defining a direct molecular mechanism, our findings identify HILPDA as a hypoxia-associated gene consistently linked to ferroptosis-related transcriptional programs across bulk and single-cell transcriptomic datasets. This integrative approach provides a hypothesis-generating model for future functional studies investigating the hypoxia–ferroptosis relationship in glioblastoma.

## 4. Material and Methods

### 4.1. Data Source and RNA-Seq Preprocessing

In this study, RNA-seq gene expression data and clinical information for patients with glioma were obtained from The Cancer Genome Atlas (TCGA) database [18,19]. Analyses were performed separately on the TCGA-LGG and TCGA-GBM cohorts and validated in a combined glioma cohort (GBM+LGG). Transcriptome data were downloaded from the GDC Data Portal [20] under “Transcriptome Profiling-Gene Expression Quantification” using the HTSeq-STAR Counts workflow, which is based on the STAR aligner [21] and the HTSeq quantification framework [22]. Analyses were conducted separately for the TCGA-LGG and TCGA-GBM cohorts and subsequently validated in a combined glioma cohort (GBM+LGG). Clinical data were obtained separately on a project basis and matched on a sample basis using the first 16 characters of the TCGA barcodes. Only matched samples were included in the analysis by considering the intersection between the expression matrix and clinical picture. Duplicate samples and cases with missing clinical data were excluded. The raw count matrix was analyzed using the DESeq2 package (v1.38.0) in R (v4.2.2) [23]. To stabilize technical variation between samples, a variance-stabilizing transformation (VST) was applied, and the VST-normalized continuous expression matrix was used in all downstream analyses. Ensembl gene identities were converted to HGNC gene symbols using the biomaRt package (v2.54.0) [24], querying the Ensembl database (release 109, https://www.ensembl.org) [25]. All analyses were conducted in R (v4.2.2). If multiple transcripts corresponded to the same gene symbol, the representative with the highest variance was selected. Genes with undefined or NA symbols were excluded from the analysis.

### 4.2. Calculation of Hypoxia and Ferroptosis-Related Gene Signature Scores (GSVA)

Gene Set Variation Analysis (GSVA) was applied to estimate sample-level pathway enrichment scores [26]. The hypoxia score was calculated using the Hallmark Hypoxia gene set obtained from the MSigDB database [27]. The ferroptosis score (FerroScore) was calculated using a curated ferroptosis-related gene set derived from the FerrDb database, a manually curated repository of ferroptosis regulators and markers [28], and supported by established literature describing key ferroptosis-related genes [4,5,29]. The gene set included ferroptosis-related genes involved in iron metabolism, lipid peroxidation, and antioxidant defense, namely SLC7A11, GPX4, ACSL4, TFRC, FTH1, FTL, HMOX1, NFE2L2, ALOX15, and SAT1. Importantly, this gene set includes both positive and negative regulators of ferroptosis; therefore, the resulting FerroScore does not represent direct ferroptotic activity but rather reflects the overall ferroptosis-related transcriptional signature of each sample. The ferroptosis gene set was curated from the FerrDb database and included both ferroptosis driver and suppressor genes. Therefore, the ssGSEA-derived FerroScore represents the overall enrichment of ferroptosis-related transcriptional programs rather than ferroptosis activation alone. GSVA analyses were performed using the GSVA package (v1.46.0) in R (v4.2.2) with the kcdf = “Gaussian” parameter, as a normalized continuous expression matrix was used [26]. Enrichment scores calculated for each sample were defined as the hypoxia score and FerroScore, respectively. Scores were divided into two groups based on the median value: High (≥median) and Low (<median). This binary classification was used as a grouping variable in downstream differential expression, immune infiltration, and survival analyses.

### 4.3. Differential Gene Expression Analysis

Differential gene expression analysis was performed between the hypoxia-high and hypoxia-low groups and as well as between the ferroScore-high and ferroScore-low groups. A DESeq2 model was constructed using the group variable within a generalized linear model framework based on the negative binomial distribution [23]. Contrast analysis was applied to estimate log2 fold changes between predefined groups. Statistical significance was determined using the Benjamini–Hochberg procedure to control the false discovery rate (FDR) [30]. Genes were considered significantly differentially expressed according to the following threshold criteria: |log2 Fold Change| > 1 and adjusted *p*-value (FDR) < 0.05. Genes meeting these criteria were defined as biologically significant differentially expressed genes (DEGs).

### 4.4. Heatmap Analysis of 12 Genes Associated with Hypoxia

Expression patterns were evaluated for 12 selected hypoxia associated genes (NGB, ALOX12B, ATP6V1G2, GDF15, DPP4, HILPDA, VEGFA, CA9, PLIN2, NNMT, IL6, and HMOX1). Hypoxia-High and Hypoxia-Low groups were created using median splitting based on hypoxia scores. Gene expression values were subjected to Z-score standardization on a row basis (mean = 0, sd = 1 for each gene). A heatmap was created using the ComplexHeatmap package (v2.14.0) in R (v4.2.2) [31]; genes were in the rows, and samples were in the columns. The columns were sorted according to the hypoxia group, and hypoxia status is shown in the upper annotation bar.

### 4.5. Gene-Based Survival Analysis

Overall survival (OS) analysis was performed using the survival (v3.5-5) and survminer (v0.4.9) packages in R (v4.2.2). OS duration was calculated from the clinical table in days and converted to the year format in the graphs. The event variable was defined according to vital status information (death = 1, survival = 0). For each gene, patients were divided into high- and low-expression groups according to the median expression of the relevant gene. Kaplan–Meier curves were created [32], and the difference between the groups was evaluated using the log-rank test [33].

### 4.6. Immune Signature Analysis (ssGSEA)

The ssGSEA method was applied to evaluate immune activity in the tumor microenvironment [34]. The following immune signature sets were used: CD8^+^ T cells, cytolytic activity, IFN-γ response, NK cells, macrophage signature, and Treg signature. Enrichment scores were calculated for each sample using the GSVA (v1.46.0) in R (v4.2.2) [26]. Scores between the HILPDA-High and HILPDA-Low groups, created by median split according to HILPDA expression, were compared using the Wilcoxon rank-sum test [35]. Visualization was performed using a combination of violin plot + boxplot + jitter, with an independent *y*-axis in each panel and automatic *p*-value annotation.

### 4.7. Correlation Analysis

The relationship between HILPDA expression and immune parameters (CD8 score, IFN-γ response, cytolytic score, and PD-L1 expression) was evaluated using Spearman ’s rank correlation analysis [36]. Correlation coefficients (ρ) and *p*-values were calculated; regression lines and 95% confidence intervals are shown in the scatter plots.

### 4.8. Survival and Cox Regression Analyses

Differences in survival between the high- and low-risk groups, according to hypoxia and ferroptosis scores, were analyzed using the Kaplan–Meier method [32]. Group differences were assessed using the log-rank test [33].

To further assess the prognostic impact of hypoxia and ferroptosis scores, univariate and multivariate Cox proportional hazards regression analyses were performed [37]. Clinical covariates, including age and sex, were incorporated into the multivariate model to evaluate the independent prognostic significance of molecular risk groups. Hazard ratios (HRs) and corresponding 95% confidence intervals (CIs) were reported.

### 4.9. Single-Cell RNA-Seq Analysis (GSE131928)

Single-cell RNA sequencing data from human glioblastoma samples were downloaded from the Gene Expression Omnibus (GEO) database with accession number GSE131928 [38]. IDH-wildtype glioblastoma samples from the 10X Genomics platform were used in the analyses. The raw count matrix was analyzed using the Seurat package (v5) in R (v4.5.1) [39,40]. A Seurat object was created using the count data, and cells with low gene counts and potentially low-quality cells were filtered out during the quality control stage. The data were normalized using log-normalization, variable genes were identified, and the data were scaled. Principal component analysis (PCA) was first applied for dimensionality reduction, followed by UMAP using the first 20 components [41]. The intercellular distribution of HILPDA gene expression was visualized using the FeaturePlot function on UMAP. To verify cell types, immune cell markers (PTPRC, LYZ, and C1QA) and malignant cell markers (EGFR, SOX2, and OLIG2) were examined. To define the malignant cell population, cells showing high expression of PTPRC, LYZ, and C1QA were considered immune cells and excluded from the analysis. The remaining cells were evaluated as malignant. Hypoxia and ferroptosis pathway activities were quantified at the single-cell level using the AddModuleScore function implemented in Seurat [42]. The Hallmark Hypoxia gene set from MSigDB [27] was used to calculate hypoxia activity, while ferroptosis activity was computed using a curated gene list derived from FerrDb [28]. The resulting HypoxiaScore and FerroScore values were incorporated into the cell metadata. The relationship between HILPDA expression and hypoxia and ferroptosis scores was evaluated using Spearman’s correlation analysis [36]. Correlation analyses were performed separately for both the entire cell population and only for the malignant cell subset.

### 4.10. Statistical Analysis

Multiple testing correction was performed using the Benjamini–Hochberg false discovery rate (FDR) method [30]. Statistical significance was set at *p* < 0.05. All analyses were performed in R (v4.5.1 (*R: The R Project for Statistical Computing*, n.d.).

## 5. Conclusions

In conclusion, this study presents an integrative transcriptomic analysis linking hypoxia signaling, ferroptosis-related gene expression signatures, and immune microenvironment remodeling in glioblastoma. Our bulk and single-cell transcriptomic analyses consistently identify HILPDA as a hypoxia-associated gene linked to ferroptosis-related transcriptional programs and immune-related signatures. These findings suggest a transcriptomic association between hypoxia signaling and ferroptosis-related gene expression patterns rather than a direct mechanistic regulatory role. Therefore, this study should be considered hypothesis-generating and provides a framework for future experimental studies investigating the functional role of HILPDA in the hypoxia-ferroptosis relationship. Our single-cell and mediation analyses suggest that the relationship between HILPDA and ferroptosis-related gene expression is likely indirect and mediated through hypoxia-related transcriptional programs rather than a direct ferroptosis-regulatory mechanism.

## Data Availability

Data supporting the findings of this study are available from the corresponding author upon reasonable request.

## Supplementary Materials

The following supporting information can be downloaded at https://www.mdpi.com/article/10.3390/ijms27083698/s1.
