# Supplementary material for: Integrated Transcriptomic and Single-Cell Analyses Identify HILPDA as a Hypoxia-Mediated Regulator of Ferroptotic Signaling in Glioblastoma
Source: Int J Mol Sci. 2026 Apr 21;27(8):3698. doi: 10.3390/ijms27083698 (PMC13116775; doi:10.3390/ijms27083698)
Supplement: Supplementary file 1 [file ijms-27-03698-s001.zip › ijms-4209695-supplementary.pdf]

Supplementary Table S1. Multivariate Cox regression analysis in the TCGA-GBM cohort

Variable	HR	95% CI	p-value
**Age (years)**	1.00	1.00–1.00	0.0004
**Sex (Male vs Female)**	1.49	1.10–2.03	0.010
**IDH status (Wild-type vs Mutant)**	2.99	1.48–6.09	0.002
**MGMT promoter (Unmethylated vs Methylated)**	1.36	1.01–1.84	0.043
**HILPDA expression**	1.00	1.00–1.00	0.089
